# Perioperative Use of Pulmonary Artery Catheter in a Patient With Severe Chronic Thromboembolic Pulmonary Hypertension Undergoing Major Gynecological Surgery: A Case Report

**DOI:** 10.7759/cureus.103094

**Published:** 2026-02-06

**Authors:** Laura Kekec, Sanja Radisavljević Vitas, Mojca Bervar, Gordana Taleska Štupica

**Affiliations:** 1 Faculty of Medicine, University of Ljubljana, Ljubljana, SVN; 2 Clinical Department of Anaesthesiology and Perioperative Intensive Therapy, University Medical Centre Ljubljana, Ljubljana, SVN; 3 Clinical Department of Cardiology, University Medical Centre Ljubljana, Ljubljana, SVN

**Keywords:** anaesthesia, chronic thromboembolic pulmonary hypertension (cteph), noncardiac surgery, pulmonary artery catheter, pulmonary hypertension

## Abstract

Pulmonary hypertension (PH) is associated with substantial perioperative risk in noncardiac surgery. In chronic thromboembolic pulmonary hypertension (CTEPH), persistent elevation of pulmonary vascular resistance (PVR) imposes considerable mechanical load on the right ventricle (RV), limiting its ability to tolerate acute physiologic changes. We report the successful management of a 52-year-old woman with severe CTEPH undergoing total hysterectomy and bilateral salpingo-oophorectomy. Comprehensive preoperative assessment, meticulous intraoperative management, and continuous hemodynamic monitoring with a pulmonary artery catheter (PAC) enabled tight control of pulmonary pressures without vasopressor support. Under low-dose glyceryl trinitrate infusion and PAC-guided titration of therapy, her mean pulmonary artery pressures (mPAP) remained stable throughout surgery and the early postoperative period. She experienced an uneventful recovery and was discharged home on postoperative day eight. This case underscores the critical role of meticulously tailored anesthetic management and advanced monitoring (PAC) in ensuring optimal outcomes for patients with severe PH undergoing noncardiac surgery.

## Introduction

Pulmonary hypertension (PH) is consistently identified as a major risk factor for perioperative morbidity, especially in patients undergoing moderate-to-high risk noncardiac surgeries. Mortality rates in PH populations can approach 18%, reflecting the potential for right ventricular (RV) decompensation and hemodynamic instability under the stresses of anesthesia, surgical manipulation, and fluid shifts [1,2].

Chronic thromboembolic pulmonary hypertension (CTEPH) arises from organized clots obstructing the pulmonary vasculature, leading to chronically elevated pulmonary vascular resistance (PVR) and progressive RV maladaptation [2]. Anesthetic challenges in CTEPH include preventing abrupt increases in PVR (e.g., hypoxia, hypercarbia, pain, airway stimulation), maintaining RV perfusion despite anesthesia-induced reductions in systemic vascular resistance (SVR), and selecting an anesthetic technique appropriate for patient physiologic status and surgical requirements. Although both transthoracic (TTE) and transesophageal echocardiography (TEE) provide essential episodic evaluations of RV size and function, a pulmonary artery catheter (PAC) allows continuous real-time measurement of pulmonary pressures, cardiac output, and mixed venous oxygen saturation, as a direct and global surrogate marker for the adequacy of tissue perfusion.

In this report, we describe the perioperative management of a 52-year-old female patient diagnosed with severe CTEPH who underwent a total hysterectomy and bilateral salpingo-oophorectomy for large uterine masses. We highlight the rationale for using a PAC, employing meticulous anesthetic strategies, and administering carefully titrated intravenous glyceryl trinitrate to optimize pulmonary hemodynamics. Written informed consent for publication of this case report was obtained from the patient.

## Case presentation

Initial presentation and workup

**Figure 1:**
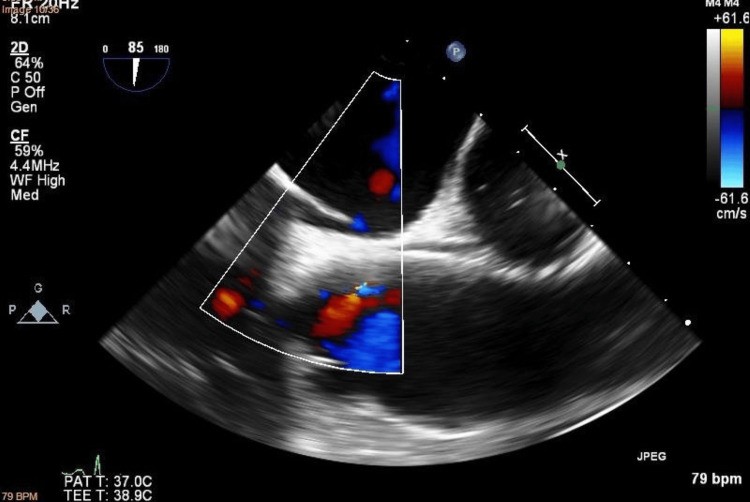
No signs of patent foramen ovale on transesophageal echocardiography with color Doppler

**Figure 2:**
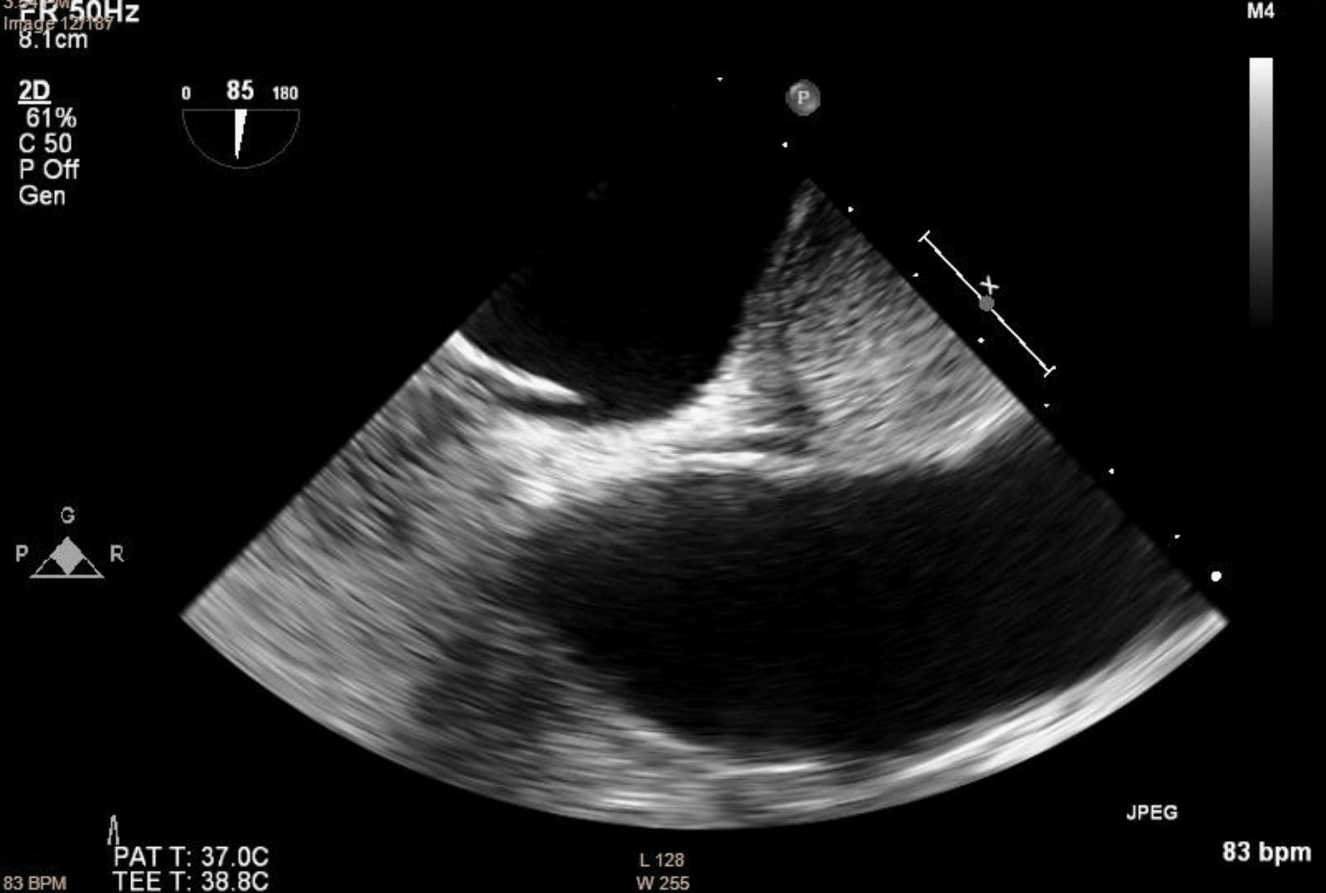
Transesophageal echocardiography of interatrial septum during bubble study: no signs of passing bubbles through the interatrial septum

A 52-year-old female patient with a long-standing history of smoking was admitted to the hospital for the acute onset of speech difficulties and right-sided motor deficits. Brain imaging revealed multiple areas of encephalomalacia from previous ischemic events, along with a new insult in the left lentiform nucleus. Vascular imaging of the neck showed no abnormalities. During her inpatient stay, she received acetylsalicylic acid, enoxaparin, and rosuvastatin, which led to gradual improvement in her neurologic function. A transcranial Doppler study with Valsalva maneuver identified numerous microemboli and suggested a right-to-left shunt, although subsequent TEE did not confirm a patent foramen ovale (Figures 1-2).

Initial echocardiography demonstrated mildly reduced left ventricular systolic function (LVEF) of 50%, left ventricular outflow tract velocity time integral (LVOT VTI) of 16.1, a mildly enlarged right ventricle (diameter at end-diastole, 4.4 cm), and reduced RV systolic function, with tricuspid annular plane systolic excursion (TAPSE) of 1.4 cm and RV systolic excursion velocity by tissue Doppler (S') of 6 cm/s. Doppler assessment demonstrated echo-derived estimates of severe PH: systolic pulmonary artery pressure (sPAP) ≈ 68 mmHg, mean pulmonary artery pressure (mPAP) ≈ 41 mmHg, and PVR ≈ 5 Wood units. PVR was estimated by echocardiography using the ratio of tricuspid regurgitation velocity to right ventricular outflow tract velocity-time integral. A chest computed tomography angiogram (CTA) revealed chronic bilateral chronic pulmonary emboli accompanied by right heart strain, while lower-extremity Doppler detected both acute and chronic popliteal deep vein thromboses. Given these findings, acetylsalicylic acid was discontinued, and the patient was placed on therapeutic doses of dalteparin. Workup for antiphospholipid syndrome and Sjögren syndrome was initiated but not completed prior to discharge.

**Figure 3:**
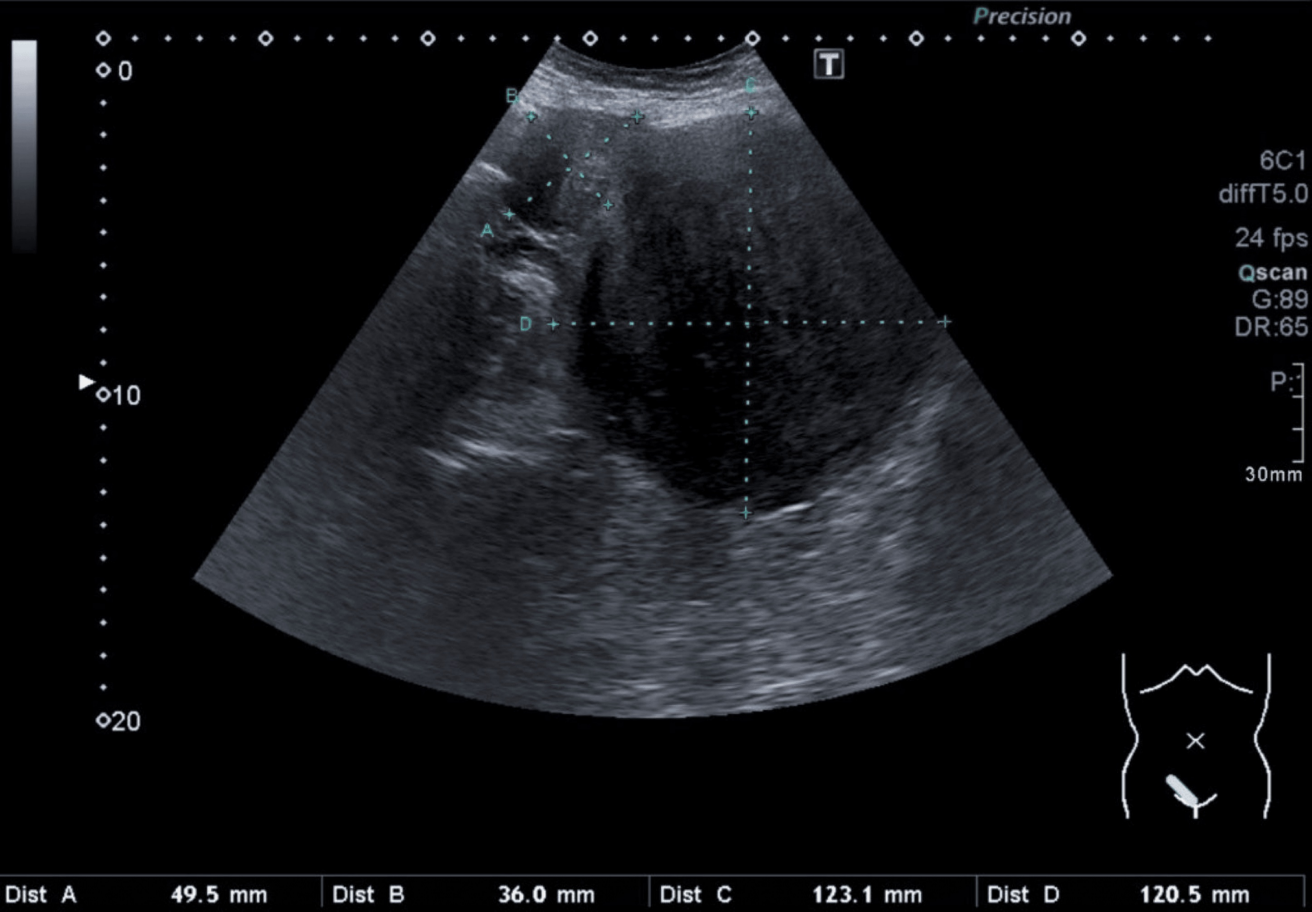
An abdominal ultrasound of the big tumorous mass in the uterus and the smaller mass next to it

During her hospital course, an abdominal mass was noted on physical examination. Ultrasound showed an enlarged uterus of 15 × 12 cm with an additional solid lesion of 5 × 3.5 cm (Figure 3). The gynecology team recommended a total hysterectomy and bilateral salpingo-oophorectomy via a lower median laparotomy. To ensure medical stability after her recent cerebrovascular event, the operation was deferred until five months later. An inferior vena cava filter was placed one week before the planned surgery to minimize further pulmonary embolic risk.

Preoperative status

**Figure 4:**
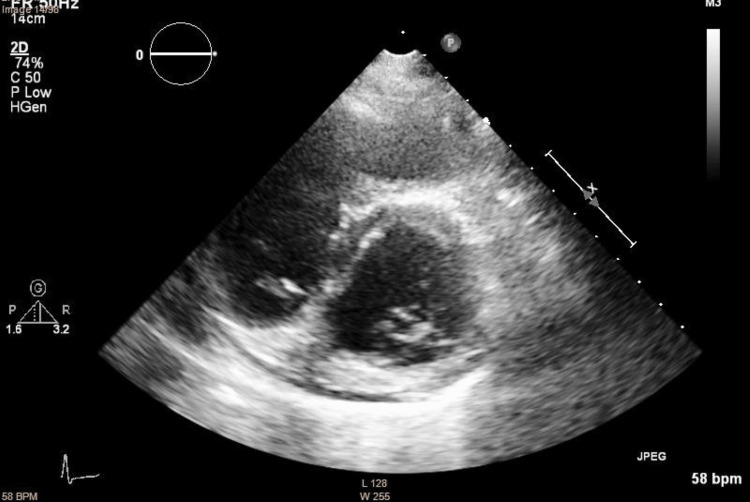
Larger right ventricle and D-sign, seen on parasternal short axis

**Figure 5:**
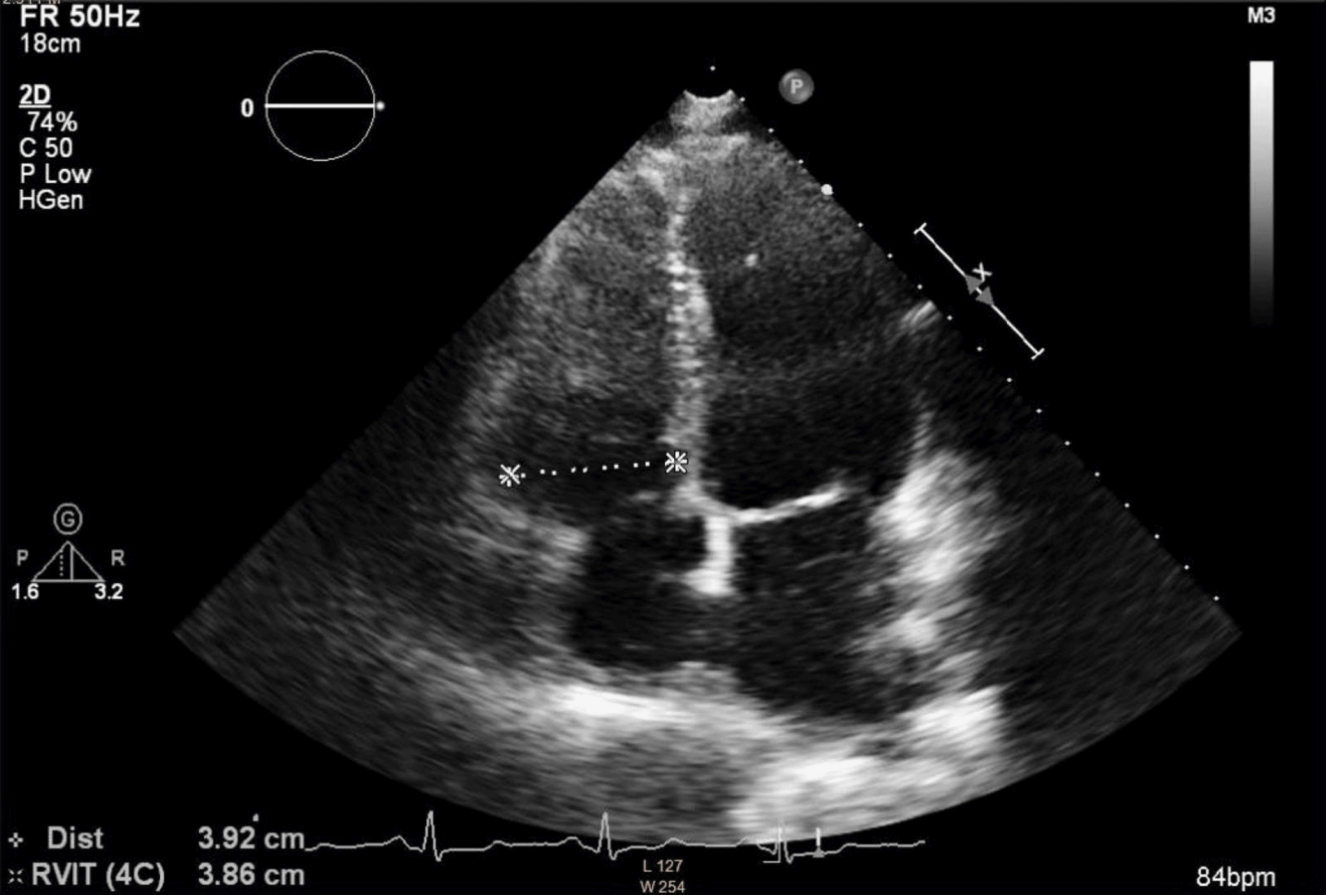
Right ventricle at the upper limit of normal size, assessed in the apical four-chamber view

**Figure 6:**
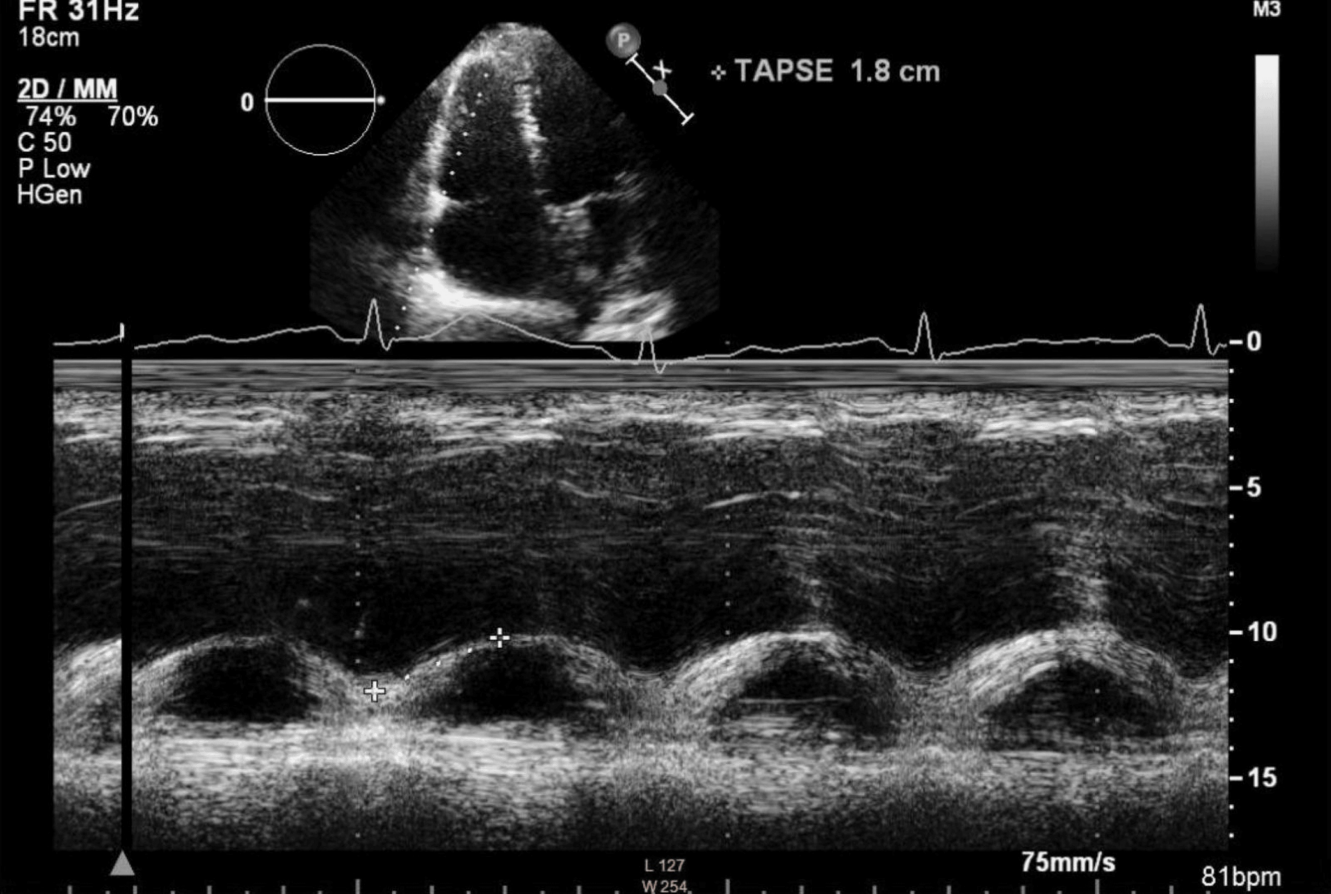
Measurement of TAPSE. TAPSE calculation in M-mode, using the four-chamber view TAPS: tricuspid annular plane systolic excursion The value (1.8 cm) has improved compared to the initial examination (1.3 cm); however, it remains at the lower limit of normal

**Figure 7:**
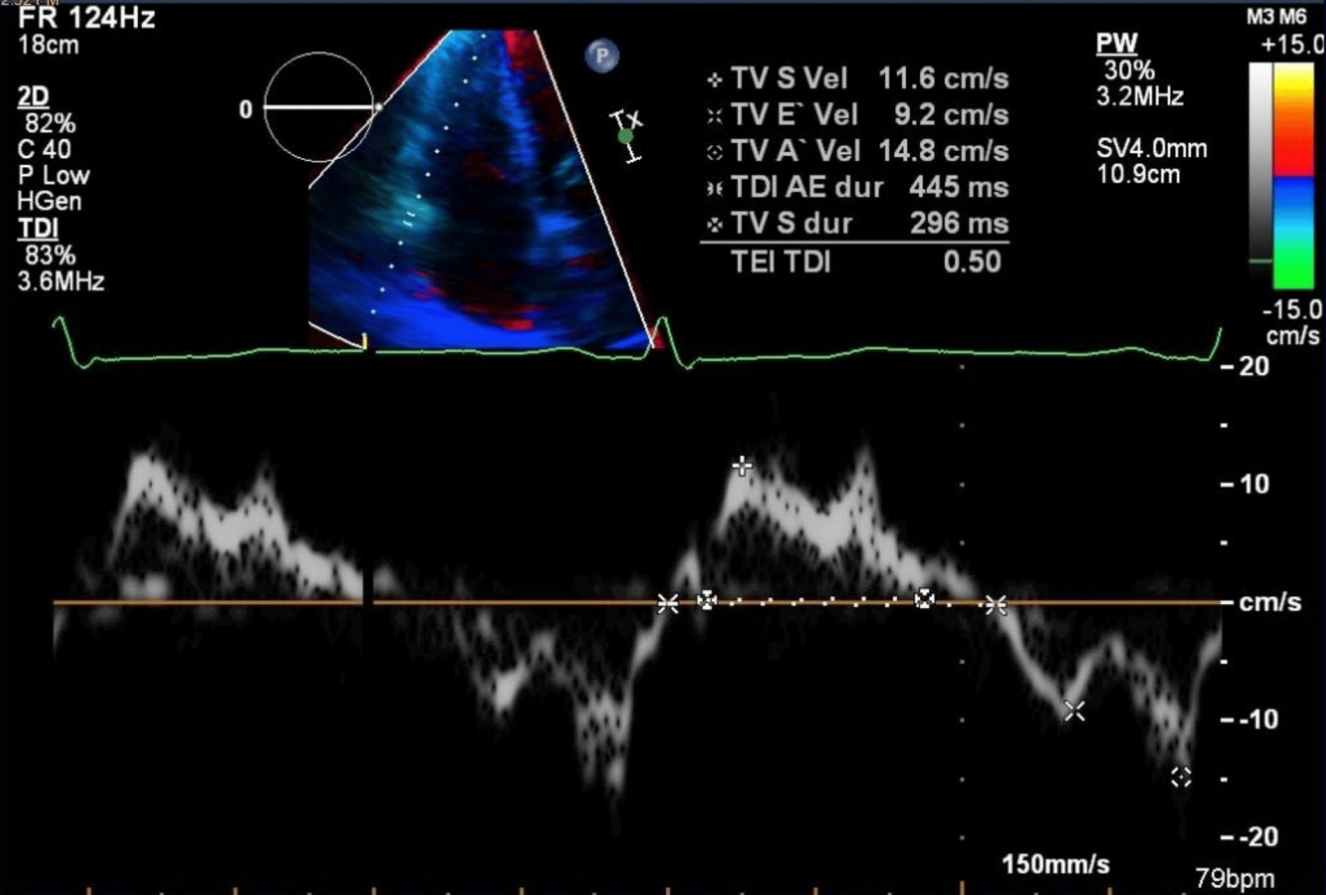
Pulsed-wave TDI of the lateral tricuspid annulus in an apical four-chamber view TDI: tissue Doppler imaging; S': systolic; E': early diastolic; A': late diastolic; TV: tricuspid valve The spectral Doppler trace displays the S’, E’, and A’ annular velocities, reflecting right ventricular longitudinal myocardial function. The measured velocities include TV S’ 11.6 cm/s, TV E’ 9.2 cm/s, and TV A’ 14.8 cm/s, respectively. Time intervals for isovolumic contraction, ejection, and relaxation are shown, allowing calculation of the right ventricular myocardial performance index (Tei index = 0.50). These measurements correspond to borderline-normal systolic function of the right ventricle

**Figure 8:**
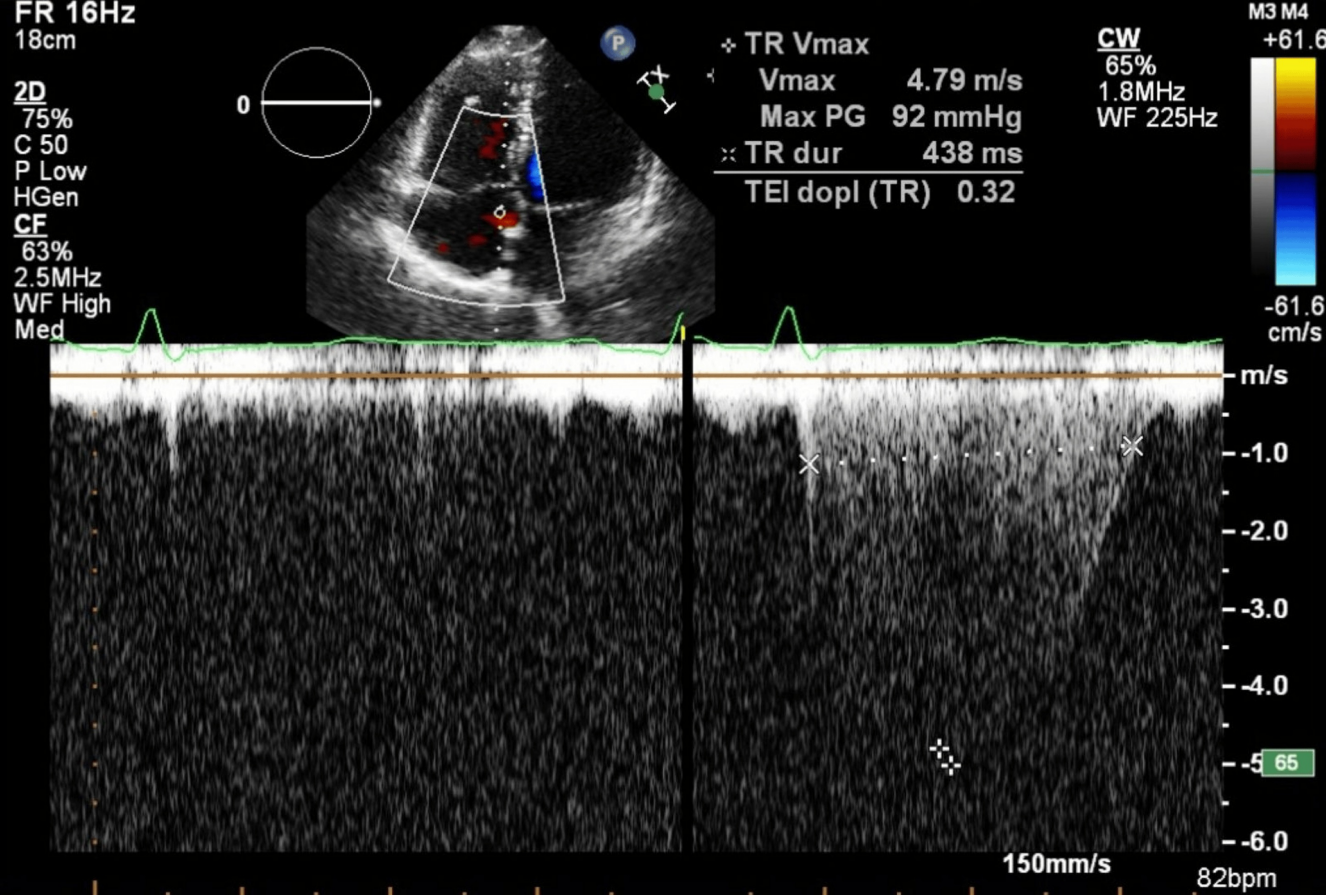
Apical four-chamber view with color Doppler demonstrating a TR jet, with CW Doppler aligned through the regurgitant flow CW: continuous wave; TR: tricuspid regurgitation The CW Doppler spectrum shows a peak TR velocity (Vmax) of 4.79 m/s, corresponding to a calculated peak systolic transtricuspid pressure gradient of 92 mmHg (modified Bernoulli equation). Tricuspid regurgitation duration is 438 ms, and the Doppler-derived Tei index from the TR signal is 0.32, indicating preserved to mildly impaired global right ventricular function despite markedly elevated right ventricular systolic pressure, consistent with compensated pressure overload. Measurements were obtained from an incomplete jet due to a limited acoustic window and represent the best achievable measurement and should be interpreted as an estimated value

**Figure 9:**
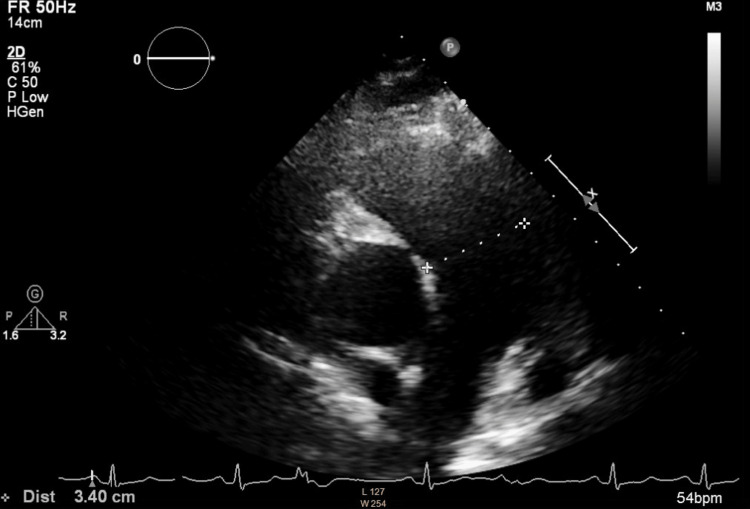
Enlarged pulmonary arteries are seen on the parasternal short-axis view, with the main pulmonary artery measuring 3.4 cm in diameter

The patient was classified as American Society of Anesthesiologists (ASA) Physical Status 4 and New York Heart Association (NYHA) Class II. She remained clinically stable, with no evidence of edema, normal pulmonary auscultation, and no signs of hepatomegaly. Her blood pressure measured 123/77 mmHg, heart rate 65 beats per minute, and oxygen saturation remained at 100%. Laboratory results revealed an NT-proBNP of 957 ng/L and a fibrinogen level of 7.78 g/L, but were otherwise unremarkable. Pulmonary function testing showed mild obstructive ventilatory physiology (FVC, 89%; FEV₁, 79%; FEV₁/FVC, 70%). A follow-up echocardiographic examination was performed prior to the procedure. Compared with the initial study, it demonstrated a modest improvement. The examination showed a normal systolic function of the left ventricle, a right ventricle at the upper limit of normal size with diameter at end-diastole of 3.8 cm (Figures 4, 5), systolic function of the right ventricle at the lower limit of normal with TAPSE of 1.8 cm (Figure 6), without diastolic dysfunction (Figure 7), and persistent severe PH, with maximal tricuspid regurgitation (TR) pressure gradient of 92 mmHg (Figures 8, 9). Two units of packed red blood cells were cross-matched in case transfusion became necessary. The final dose of dalteparin (5,000 IU) was administered 12 hours before the operation to reduce perioperative bleeding risks while addressing her thromboembolic history.

Intraoperative course

Upon arrival in the operating room, the patient had an intravenous line and an arterial line inserted for continuous blood pressure monitoring. Her initial blood pressure was 140/82 mmHg, heart rate was 65 beats per minute, and oxygen saturation was 100%. After receiving standard intravenous prophylactic antibiotics, she underwent induction of general anesthesia with 0.2 mg of fentanyl, 14 mg of etomidate, and 40 mg of rocuronium. Etomidate was selected due to its stable hemodynamic profile, which is particularly suitable in cases of severe PH [3]. Following endotracheal intubation, anesthesia was maintained with sevoflurane at approximately 2% end-tidal concentration, supplemented with additional fentanyl and rocuronium boluses as required.

Shortly after induction, an infusion of glyceryl trinitrate was initiated at 2 mg/h, not as targeted PH therapy, but to modulate systemic blood pressure and venous return, reducing RV wall stress. To enable detailed hemodynamic assessment, the anesthesiology team placed a central venous catheter (CVC) for fluid and drug administration, as well as a Swan-Ganz PAC to continuously monitor right-sided filling pressures and pulmonary artery pressures. Over the course of the 50-minute surgical procedure, mPAP remained between 28 and 35 mmHg, and central venous pressure (CVP) stayed in the range of 6 to 9 mmHg. After induction of anesthesia, her systolic blood pressure was consistently maintained between 110 and 120 mmHg, mean arterial pressure (MAP) between 70 and 80 mmHg, heart rate around 70 beats per minute, and oxygen saturation was consistently 98-100%. No vasopressors were required. Ventilatory parameters were set to deliver tidal volumes of 6-8 mL per kg of ideal body weight, along with a positive end-expiratory pressure (PEEP) of 5 cmH2O, to maintain normocapnia and minimize increases in intrathoracic pressure. Blood loss was minimal, and no transfusions were necessary. In addition, antiemetics and nonopioid analgesics (metamizole) were administered to prevent nausea and to mitigate pain and sympathetic responses that could have elevated pulmonary pressures.

Postoperative management

At the end of surgery, the patient was extubated in the operating room and transferred to an intermediate care setting for close observation. She continued receiving glyceryl trinitrate at 0.5-1 mg/h to stabilize afterload and preload; mPAP remained in the 27-35 mmHg range during overnight monitoring through the PAC. On postoperative day one, with no signs of right ventricular failure or systemic hypotension, the glyceryl trinitrate infusion was discontinued, and the PAC was removed. Pain management included a patient-controlled analgesia (PCA) pump delivering piritramide, along with intravenous paracetamol and metamizole. Throughout this period, pain scores did not exceed 4 out of 10, and the patient required no vasopressor or pulmonary vasodilator support. The central venous catheter was removed on day four, and laboratory tests showed only a transient mild hypoalbuminemia. Hemoglobin levels never fell below 100 g/L, eliminating any need for transfusion. Mobilization began on postoperative day six without complications, and on postoperative day eight, she was discharged home on her previous regimen of dalteparin and rosuvastatin, having recovered uneventfully from both her surgery and her underlying severe PH.

## Discussion

Patients with severe PH face increased perioperative risk due to the right ventricle’s inability to tolerate abrupt fluctuations in loading conditions [2,4]. CTEPH adds a permanent elevation of PVR from unresolved thrombi, placing significant mechanical strain on the RV [5,6]. Even moderate-risk surgeries can prove hazardous when the RV is already compromised, emphasizing the need for individualized strategies and specialized monitoring.

A crucial consideration is comprehensive preoperative assessment. Detailed TTE helps characterize RV size, wall thickness, and systolic function (e.g., via TAPSE), while also estimating pulmonary pressures [7,8]. Identifying any mechanical lesions (e.g., patent foramen ovale, valvular dysfunction) and evaluating functional capacity (NYHA classification, six-minute walk test, or cardiopulmonary exercise testing) further refines risk stratification [2,7]. In this patient, although a cardiopulmonary exercise test was not performed, the borderline RV function and persistently high estimated pulmonary pressures made it clear that advanced support, including continuous invasive monitoring, could become critical. Pulmonary pressures and PVR were echo-derived estimates, consistent with current echocardiographic practice.

Intraoperative success relies on the appropriate choice of anesthetic agents, guided by the principle of maintaining RV stability and avoiding large swings in SVR or PVR. Etomidate is recognized for providing stable hemodynamics at induction in PH patients, though repeated doses can cause adrenal insufficiency [3,9]. Alternatively, agents like ketamine might preserve systemic pressure but occasionally elevate PVR [10,11]. Propofol, widely used for induction, often lowers blood pressure by reducing SVR, which may compromise coronary perfusion of the hypertrophied RV if not carefully balanced. Selecting sevoflurane for maintenance anesthesia can also be beneficial, as it generally has modest effects on PVR if normocapnia and adequate oxygenation are maintained [12,13]. Maintenance of anesthesia can be achieved with any inhalational agent except nitrous oxide, which increases PVR [7]. Whenever feasible, regional anesthesia and epidural anesthesia are recommended for CTEPH patients [7,9]. In the case of our patient, intraoperative anesthesia could potentially have been achieved with a high spinal block. However, in this particular patient, such an approach carried a significant risk of hemodynamic instability due to sympathetic blockade at the thoracic level and was therefore not considered a viable option.

The use of vasodilator therapy to manage PH in the perioperative setting is well-documented. Authors have described intravenous or inhaled agents like nitric oxide, iloprost, milrinone, and prostacyclin analogs to mitigate high pulmonary pressures [9,11,12,14]. As pulmonary pressures remained stable, targeted pulmonary vasodilator therapy was not required in our patient. Instead, our patient received a glyceryl trinitrate infusion to modestly decrease preload and afterload, thus reducing RV strain. Although systemic vasodilation always risks hypotension, we avoided that complication by tightly controlling the infusion rate and maintaining systolic blood pressure between 100 and 130 mmHg, and mean systolic blood pressure between 70 and 80 mmHg. This approach protected the coronary perfusion of the RV. Maintaining adequate MAP is essential because the RV is susceptible to ischemia if coronary blood flow declines, especially when facing elevated pulmonary pressures [2,10].

Ventilatory management must also be carefully tailored in severe PH. Elevated intrathoracic pressures can significantly impede venous return and further increase RV afterload [7]. Strategies that limit plateau pressure and use only moderate PEEP (5-8 cmH2O) reduce the likelihood of exacerbating PVR [7]. Hypoxia and hypercapnia both intensify pulmonary vasoconstriction, emphasizing the importance of maintaining normocapnia and a sufficient fraction of inspired oxygen (FiO2). Many sources advocate for lung protective ventilation strategies with low tidal volumes (6-8 mL/kg ideal body weight) to diminish alveolar overdistension, thus safeguarding the RV [2,4,6]. PVR and pulmonary pressures increase at low lung volumes due to reduced tension in the extra-alveolar arterioles, and at high lung volumes as a result of compression of the alveolar capillaries [7].

TEE is a useful intraoperative tool for real-time assessment of RV and biventricular function and volume status. The mid-esophageal four-chamber view allows evaluation of RV size and septal motion, with diastolic leftward septal shift indicating volume overload and systolic shift suggesting pressure overload, often resulting in a “D-shaped” left ventricle [8]. TEE can also estimate sPAP by calculating RV systolic pressure from right atrial pressure and the TR gradient, making it valuable for hemodynamic monitoring during major, prolonged, or high-risk surgeries under general anesthesia [7]. An especially important element in this case was the decision to use a PAC. While TEE can provide intermittent functional data (e.g., RV ejection fraction, volume status, valvular lesions) [14], it generally requires an advanced operator for repeated measurements and might be less practical for continuous perioperative-to-postoperative transitions, particularly in noncardiac units without immediate echocardiographic expertise [5,10]. The PAC offers real-time measurement of mPAP, cardiac output, and mixed venous oxygen saturation (SvO2), enabling immediate titration of fluid therapy, vasodilators, or inotropes if the RV begins to fail [2,8]. Such real-time monitoring is invaluable when the margin for error is narrow, as in severe CTEPH, where a sudden rise in PVR or drop in cardiac output can precede rapid decompensation. Although potential complications like arrhythmias, infection, and pulmonary artery rupture exist, the benefits can outweigh these risks in experienced hands, and data suggest that routine complications are relatively rare if the catheter is managed cautiously [2,6,8]. Nevertheless, the accuracy of pulmonary artery catheterization may be limited by significant tricuspid regurgitation or intracardiac shunting, which interfere with thermodilution cardiac output measurements [2,4]. In these situations, monitoring hemodynamic trends during surgery could be more informative. According to most guidelines, PAC is indicated mainly in patients with severe PH [2,7-9], whereas its benefit in low-risk noncardiac procedures for patients with mild to moderate PH is limited [2].

In addition to choosing suitable anesthetic agents and placing a PAC, hemodynamic goals revolve around maintaining a stable right atrial pressure (or CVP) of about 6-10 mmHg and limiting fluid overload. Overdistension of the RV can lead to interventricular septal shift, reducing left ventricular filling and cardiac output. Conversely, hypovolemia may exacerbate hypotension if vasodilators are being used [4,5]. Subramaniam and Yared [13] emphasize that balancing vasodilation, fluid therapy, and mild-to-moderate inotropic support is often the key to success in these challenging patients. If right heart failure becomes imminent, rescue measures can include inhaled pulmonary vasodilators or mechanical circulatory support, though these carry additional risks [2,12].

Effective pain management is also crucial. Inadequately controlled pain can provoke sympathetic overactivity, leading to increased PVR, precipitating RV decompensation, and increasing the risk of arrhythmias. In this patient, PCA with piritramide and adjunctive nonopioids (paracetamol, metamizole) maintained stable analgesia, diminishing noxious stimuli. Avoiding potent sympathetic reflexes is central to preventing RV overload and maintaining stable hemodynamics [8]. Adequate analgesia also facilitates earlier mobilization, which can improve functional recovery and reduce complications such as deep vein thrombosis or atelectasis [1,12].

The favorable postoperative outcome in this case underlines how meticulous planning, from preoperative optimization to the removal of the PAC after mPAP stabilized, enables success. The patient’s discharge on postoperative day eight reflects that high-risk PH patients can have comparable postoperative trajectories to lower-risk populations if managed proactively. Limitations to replicating such a plan elsewhere might include the availability of advanced echocardiographic or PAC expertise, institutional familiarity with intravenous or inhaled pulmonary and systemic vasodilators, and willingness to commit to extended monitoring in an intermediate or intensive care setting.

## Conclusions

This case demonstrates that patients with severe CTEPH can successfully undergo moderate-risk noncardiac surgery if comprehensive preoperative evaluations, vigilant anesthetic management, and continuous hemodynamic monitoring are employed. In this instance, the use of a PAC allowed real-time assessment and titration of vasodilator therapy, maintaining stable right-sided pressures. The patient’s smooth postoperative recovery, culminating in discharge on postoperative day eight, exemplifies how advanced perioperative strategies can be integrated to avert right ventricular decompensation.
